# Enhanced classification and identification of bacterial and viral microorganisms by integration of MALDI-TOF mass spectrometry with artificial intelligence

**DOI:** 10.1038/s41598-026-54426-y

**Published:** 2026-08-24

**Authors:** Georgios Dolias, Olga Bragina, Andres Udal, Kairit Zovo, Georgios Kirtsanis, Stefanos Vrochidis, Yevgen Karpichev, Mostafa Bentahir

**Affiliations:** 1 Information Technologies Institute, Centre for Research and Technology-Hellas (CERTH), Thermi Thessaloniki, 57001 Greece; 2 Department of Chemistry and Biotechnology, Tallinn University of Technology (TalTech), Tallinn, 12618 Estonia; 3 Department of Software Science, Tallinn University of Technology (TalTech), Tallinn, 12618 Estonia; 4 Belgian Defence Laboratories (DLD), 1800 Peutie, Brussels, Belgium; 5 Royal Military Academy, Brussels, 1000 Belgium

**Keywords:** Computational biology and bioinformatics, Microbiology

## Abstract

The accurate and rapid identification of bacterial pathogens is essential in clinical setups and medical biodefense. Matrix-Assisted Laser Desorption Ionization Time-Of-Flight (MALDI-TOF) mass spectrometry has emerged as a powerful tool for fast and reliable microbial identification. This study assesses the performance of eight Machine Learning (ML) and two Deep Learning (DL) models trained using 5-fold cross validation in classifying microorganisms in a series of experiments based on MALDI-TOF mass spectra (*n* = 255) generated internally from seven cultured bacteria and five viral agents. The results showed that up to seven models achieved consistently robust classification across various tasks, including binary classification of viruses from bacteria and Gram-positive from Gram-negative bacteria, as well as multi-class classification of individual bacterial and viral species. Subsequently, we cross-validated three from our top-performing models, Extra Trees Classifier, Support Vector Classifier and 1-D Convolutional Neural Network, on Gram type and multi-class classification of individual bacterial species against an external dataset selected from a large MALDI-TOF MS database of highly pathogenic bacteria (curated by Robert Koch Institute). Among these, the Extra Trees Classifier consistently achieved the highest average accuracy and F1-score in both Gram type classification and species-level identification, demonstrating superior generalization across datasets. Its ensemble architecture proved particularly effective in capturing subtle spectral patterns associated with microbial cell wall composition and protein expression profiles. These results underscore the strong potential of this model to enhance MALDI-TOF-based classification frameworks in clinical microbiology and biodefense applications.

**Supplementary Information:**

The online version contains supplementary material available at 10.1038/s41598-026-54426-y.

## Introduction

Biological Warfare Agents (BWA), including microbial pathogens and toxins, represent a significant threat when misused. This risk arises not only because of their potential impact on large populations, but also from their capacity to cause targeted harm, disrupt economic systems, induce psychological distress, and destabilize political structures, even at relatively small scales. The use of BWAs by terrorists or accidental biological releases adds significant complexity to the security landscape. Robust and credible response capabilities act as a powerful deterrent by reducing the impact of deliberate, accidental, or naturally occurring events^1^. A comprehensive understanding of the biological weapons that can be developed, the technologies that enable their production and dissemination, and the actors capable of deploying them is a fundamental pillar of biosecurity. It is essential to analyze potential scenarios in which biological weapons could be designed, deployed against specific targets, and to identify strategies for detecting and mitigating the illegal use of BWAs. Knowledge of detection capabilities facilitates informed discussions on biosecurity tools and enables identification of critical gaps^2–4^. Although the Biological Weapons Convention (BWC) prohibits the development and use of biological weapons, emerging technologies such as Artificial Intelligence (AI) and synthetic biology are reshaping the landscape of biological research and security^5,6^.

Subfields of AI, including Machine Learning (ML) and Deep Learning (DL), have already demonstrated positive impacts in accelerating the discovery of new antibiotics. While conventional diagnostic tests are time-consuming delaying pathogen identification, ML and DL algorithms assist in rapid pathogen detection, antimicrobial resistance prediction, and drug discovery^7^. Furthermore, ML-based decision-support systems for detecting Chemical, Biological, Radiological, and Nuclear (CBRN) incidents have become a high-priority research area^8–10^. Despite these beneficial applications, AI exhibits inherent dual-use potential^11^. The same computational tools that advance healthcare and biodefense can also be misappropriated to design novel pathogens or optimize their dissemination^12–14^. Therefore, sustained vigilance and commitment to ethical responsibility are essential to ensure that AI technologies are applied exclusively for preventing biothreats rather than for their creation or misuse.

The successful deployment of AI to counter biothreats depends on continuous learning, access to high-quality datasets, algorithmic refinement, and iterative improvement of operational practices. Simultaneously, it is imperative to evaluate the operational risks introduced by AI in the biothreat context and to develop resilient strategies for risk mitigation^15^. Current list-based approaches to biodefense are constrained by their focus on known biological agents^16–20^. Transitioning toward systems based on agent-agnostic signatures would enable detection and characterization of both familiar and emerging pathogens, thereby enhancing adaptability in response to an evolving threat landscape^21^. ML and DL can facilitate the transition by recognizing and encoding complex patterns from multimodal data sources, as demonstrated in numerous healthcare and defense applications^22^. Functionalizing ML/DL for environmental bio-detection requires a thorough understanding of current technical capabilities and limitations^23^, since the rapid progress of scientific research and its applications increasingly outpaces enabling responsible research with optimal societal benefit^24^ and governments’ abilities to pass efficient regulations to ensure safety and security^25^.

In the last two decades, since the 2002 Nobel Prize was awarded to Koichi Tanaka, MALDI-TOF MS (Matrix Assisted Laser Desorption Ionization Time-of-Flight Mass Spectrometry) has become a rapid, cost-effective and trusted methodology for the identification of biological microorganisms^26–29^, including biological threats^30^. MALDI-TOF MS is a soft ionization technique that enables rapid and reliable identification of bacterial infections by analyzing proteomic mass spectra fingerprints of bacterial pathogens. This technique has several advantages including simplicity, reproducibility and high throughput, which have enabled its widespread adoption and implementation as a reference method in clinical laboratories. As a result, MALDI-TOF MS is now commonly used to identify bacterial pathogens in clinical specimens such as blood, respiratory and urinary tract samples after pathogens have been isolated using standard cultivation methods. Following this initial step, simple protein extraction methods are applied to prepare samples for analysis with the MALDI-TOF. The advantages and versatility of MALDI-TOF MS have led to its adoption in military settings, where it is used to detect and identify biological threat agents as well as opportunistic pathogens that infect wounded and burned soldiers during military operations.

Although MALDI-TOF MS has many advantages, there are some limitations that can compromise its accuracy and reliability in identifying certain pathogens. The mass spectra databases used to identify bacterial pathogens are very often incomplete, particularly for rare and poorly characterized species. As a result, these pathogens may be underrepresented in the databases, making it challenging to identify them accurately. In addition, the technique struggles to differentiate between closely related bacterial species and subspecies. The application of MALDI-TOF for the identification of viral pathogens is also limited, mainly because of the lack of comprehensive virus-specific databases and standardized methodologies for preparing viral samples. Despite growing interest, MALDI-TOF–based viral detection remains underexplored due to challenges such as low biomass, high host background, and limited spectral databases^31^. Nevertheless, MALDI-TOF remains one of the few technologies capable of producing phenotype-level, genome-independent fingerprints suitable for agent-agnostic detection^32^. Realizing this potential requires the development of robust and transparent computational pipelines, an area where ML/DL can provide substantial added value.

The reliance on proprietary software solutions, such as those developed by Bruker Daltonics^33^, a leading provider in the MALDI-TOF MS domain, represents limitations of the MALDI-TOF approach for data analysis. These closed-source platforms restrict access to underlying algorithms, thereby constraining researchers’ ability to modify or optimize computational workflows for specific experimental requirements. In response, the scientific community has increasingly adopted open-source ML and DL frameworks to design customized pipelines for processing complex MALDI-TOF spectral data^32^. These efforts encompass advanced computational strategies, including data preprocessing, model training, and performance evaluation, aimed at enhancing the accuracy and efficiency of microbial identification.

Several authors have integrated MALDI-TOF spectral data in their studies with supervised classification algorithms to differentiate closely related microbial species^34^. Applications included pathogen identification at genus and species levels, biomarker exploitation, and antimicrobial resistance prediction. For example, *Latilactobacillus sakei* subspecies were classified using MALDI-TOF MS, where Random Forest (RF) outperformed Partial Least Squares-Discriminant Analysis (PLS-DA), Principal Component Analysis - k-Nearest Neighbors (PCA–kNN), and Support Vector Machine (SVM) models by achieving accuracy of 95.4%^35^. Similarly, RF, SVM, and Logistic Regression have been applied to classify spectra from *Brucella* species (*B. melitensis*,* B. suis*, and *B. abortus*)^36^ displaying excellent predictive performance with 100% accuracy, while kNN have been used for *Enterococcus* species discrimination resulting in high classification accuracy of 99.1% with RF^37^. To predict antibiotic resistance in clinically relevant bacteria (*Escherichia coli*,* Staphylococcus aureus*,* Klebsiella pneumoniae*, and *Pseudomonas aeruginosa*), three ML-based models were trained, namely Support Vector Classifier (SVC), RF, and XGBoost and one DL model (Multi-Layer Perceptron)^38^, with SVC achieving the highest performance in terms of multi-label classification (accuracy = 80%, weighted F1-score = 92%). XGBoost has also demonstrated reliability in *Salmonella* serotype identification among nine tested models yielding accuracy of 95% and F1-score of 82% regarding the internal validation set^39^. Beyond MALDI-TOF, Extra Trees Classifier and Ridge Classifier have been employed for microarray gene expression classification following dimensionality reduction by coupling them with PCA, where Extra Trees Classifier outperformed with accuracy of 98.33% and F1-score of 99%^40^. Regarding DL approaches, a weighted CNN architecture derived from three 1-D CNN models was proposed, achieving robust detection of ten microbial forms compared to nine ML algorithms, resulting 99.97% in accuracy, F1-score, recall and precision^41^. Furthermore, Denoising AutoEncoder (DAE) combined with SVM for *Listeria* species classification outperformed other ML and DL models under 5-fold cross-validation scoring 100% accuracy^42^. Finally, Ridge Classifier (RC) was employed to discriminate gut bacteria samples, namely *Actinobacteria*, *Bacteroidetes* and *Firmicutes*^43^.

In this study, we applied AI models to a limited internally generated MALDI-TOF MS spectra dataset to evaluate their performance in three classification tasks involving bacterial and viral agents. The top-performing models trained on the internal bacterial dataset were subsequently cross-validated for two tasks using an external MALDI-TOF MS dataset from a larger database of highly pathogenic bacteria. Furthermore, we developed a virus identification workflow that combines a streamlined extraction protocol with AI-based classification.

Key contributions of this work include:

- Culturing and preparing bacterial and viral samples to create an internal MALDI-TOF MS dataset.
- Implementing a rigorous pre-processing pipeline for MALDI-TOF mass spectra.
- Training and evaluating 10 ML/DL models for two binary classification tasks: Bacteria vs. Viruses and Gram-positive vs. Gram-negative MALDI-TOF spectra.
- Performing multi-class classification to identify seven bacterial and five viral classes.
- Cross-validating three best-performing models for Gram-positive vs. Gram-negative classification and seven-class bacterial identification using external MALDI-TOF mass spectra curated by Robert Koch Institute (RKI).
- Developing an integrated workflow for viral spectra classification and differentiation from bacterial spectra.

## Results

In the current study, we cultured seven bacterial species and generated 165 MALDI-TOF mass spectra using three different protein extraction methods following the manufacturer’s instructions. Additionally, we produced 90 spectra from five viruses using an in-house protocol resulting in a combined internal dataset of 255 spectra. The raw spectra were preprocessed using an open-source software as detailed in the Methods section.

**Fig. 1:**
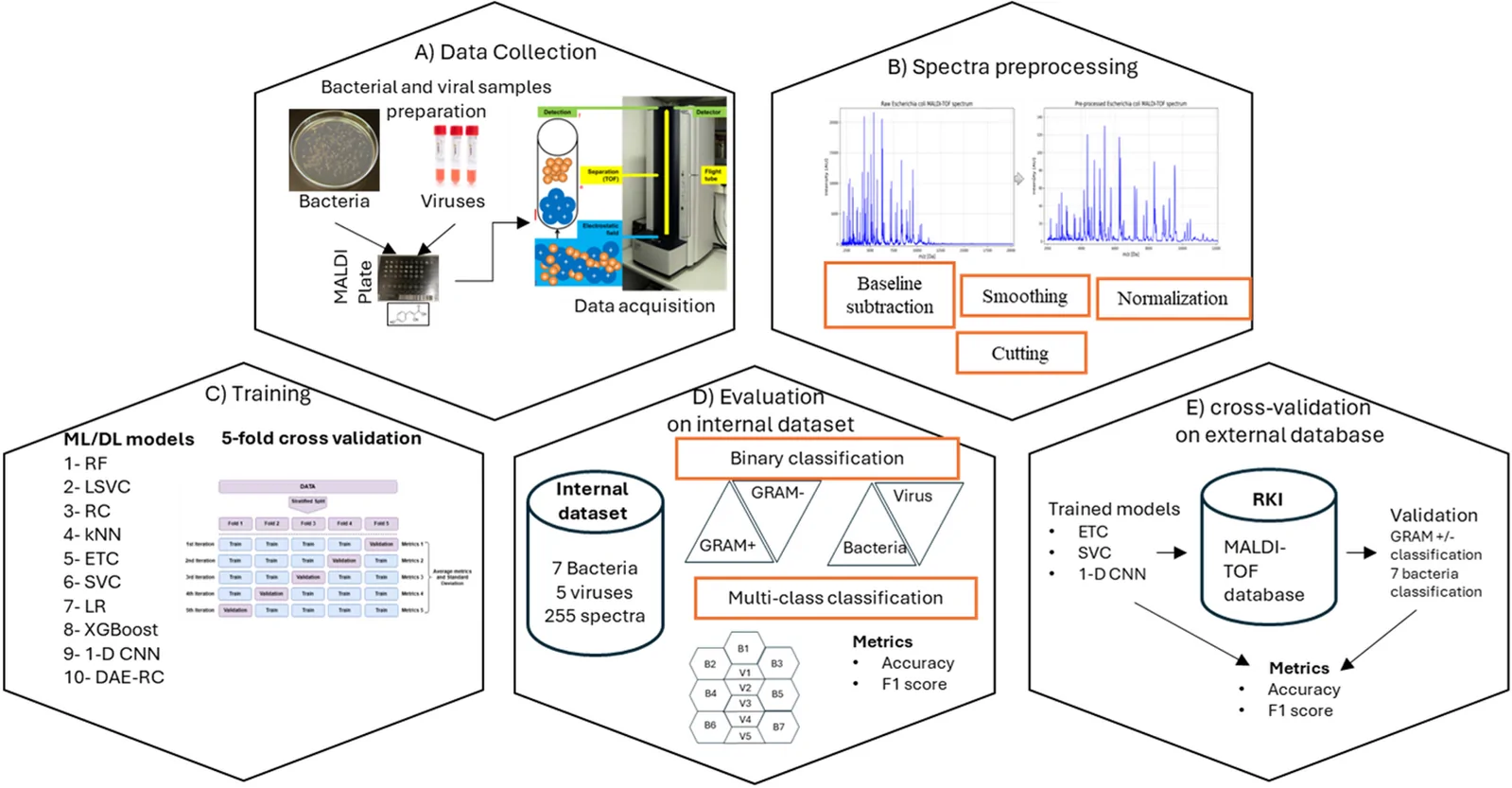
Overall workflow of our study including (A) data collection, (B) spectra preprocessing, (C) model training, (D) performance evaluation on the internal dataset using 5-fold cross validation methodology and (E) cross validation of three top-performing models on spectra from the RKI external MALDI-TOF database.

We then trained ten AI models with 5-fold cross validation and evaluated their performance across multiple classification tasks, including binary tasks (bacteria vs. virus; Gram-positive vs. Gram-negative) and multi-class microbial identification. Finally, we assessed the generalizability of three top-performing models by testing them on an external MALDI-TOF dataset from a large database of highly pathogenic bacteria, focusing on Gram type prediction and species-level identification. The overall workflow is illustrated in Fig. 1.

### Binary classification

#### Discriminating bacteria from viral bio-agents

In the initial phase of our study, we evaluated the performance of ten AI models, including eight ML and two DL models for classifying and distinguishing between two major microorganism categories: bacteria and viruses (Table 1). A dataset consisting of 255 MALDI-TOF spectra from seven bacterial species (*n* = 165) and five viral strains (*n* = 90) was preprocessed and used to train the models. As shown in Table 1, eight out of ten models achieved perfect classification performance with an average accuracy and average F1-score of 100%. The Random Forest and XGBoost models also performed strongly, though slightly lower, with average accuracy and average F1-score 99% and 98%, respectively. The low standard deviation observed across most models indicates a high consistency and reliability in classifying bacterial and viral spectra within the dataset (Table 1). These results demonstrated that MALDI-TOF mass spectra contain sufficiently distinct phenotypic signatures to robustly distinguish bacteria from viruses, underscoring the potential of this approach for rapid agent-agnostic triage in clinical and biodefense settings.

**Table 1:** Performance evaluation of 10 ML/DL models for binary classification of bacteria from viruses using the internal dataset and 5-fold cross validation.

ML/DL model	Accuracy	F1-score
Random Forest	0.99 ± 0.01	0.99 ± 0.01
Linear SVC	1.00 ± 0.00	1.00 ± 0.00
Ridge Classifier	1.00 ± 0.00	1.00 ± 0.00
kNN	1.00 ± 0.00	1.00 ± 0.00
Extra Trees Classifier	1.00 ± 0.00	1.00 ± 0.00
Support Vector Classifier	1.00 ± 0.00	1.00 ± 0.00
Logistic Regression	1.00 ± 0.00	1.00 ± 0.00
XGBoost	0.98 ± 0.02	0.98 ± 0.02
1-D CNN	1.00 ± 0.00	1.00 ± 0.00
DAE-RC	1.00 ± 0.00	1.00 ± 0.00

## Bacterial species classification based on Gram category

In a subsequent analysis, we evaluated the ability of these models to classify bacterial species based on their Gram type. We preprocessed and trained the models using 165 MALDI-TOF spectra from three Gram-positive (*n* = 71) and four Gram negative (*n* = 94) bacteria. As shown in Table 2, our findings revealed that seven models achieved perfect classification performance scores with average accuracy and average F1-score of 100%. The Random Forest, XGBoost, and DAE-RC models showed slightly lower performance with average accuracy and average F1-score remaining near perfect. Overall, the low standard deviation observed across the models indicates strong consistency and reliability in classifying bacterial spectra into their respective Gram categories (Table 2).

**Table 2:** Performance evaluation of ten ML/DL models for binary classification of Gram-positive and Gram-negative bacteria, using the internal dataset and a 5-fold cross validation approach.

ML/DL model	Accuracy	F1-score
Random Forest	0.99 ± 0.01	0.99 ± 0.01
Linear SVC	1.00 ± 0.00	1.00 ± 0.00
Ridge Classifier	1.00 ± 0.00	1.00 ± 0.00
kNN	1.00 ± 0.00	1.00 ± 0.00
Extra Trees Classifier	1.00 ± 0.00	1.00 ± 0.00
Support Vector Classifier	1.00 ± 0.00	1.00 ± 0.00
Logistic Regression	1.00 ± 0.00	1.00 ± 0.00
XGBoost	0.98 ± 0.02	0.98 ± 0.02
1-D CNN	1.00 ± 0.00	1.00 ± 0.00
DAE-RC	0.99 ± 0.10	0.98 ± 0.02

## Multi-class identification of full panel consisting of seven bacteria and five viruses

Subsequently, we evaluated the models’ ability to accurately identify the complete test panel, which included seven bacterial species and five viral strains exploiting a total of 255 MALDI-TOF mass spectra grouped into their corresponding classes. The results showed that seven models achieved perfect performance, with average accuracy and average F1-score of 100%. The KNN model also performed strongly, with scores close to 100%. In contrast, the XGBoost and DAE-RC models exhibited high F1-score, but lower average accuracy compared to the other models (Table 3). These findings underscore the robustness of most models in classifying microbial spectra while highlighting some variability in performance among specific algorithms.

**Table 3:** Performance evaluation of ten ML/DL models in 12-class classification of seven bacterial species and five viral strains using the internal dataset and a 5-fold cross validation approach.

ML/DL model	Accuracy	F1-score
Random Forest	1.00 ± 0.00	1.00 ± 0.00
Linear SVC	1.00 ± 0.00	1.00 ± 0.00
Ridge Classifier	1.00 ± 0.00	1.00 ± 0.00
kNN	0.99 ± 0.01	0.99 ± 0.01
Extra Trees Classifier	1.00 ± 0.00	1.00 ± 0.00
Support Vector Classifier	1.00 ± 0.00	1.00 ± 0.00
Logistic Regression	1.00 ± 0.00	1.00 ± 0.00
XGBoost	0.91 ± 0.01	0.91 ± 0.04
1-D CNN	1.00 ± 0.00	1.00 ± 0.00
DAE-RC	0.98 ± 0.01	0.99 ± 0.01

We next assessed the models by training them to differentiate closely related, clinically significant Gram-negative bacteria, specifically *K. aerogenes* and *K. pneumoniae*, and to distinguish these from other *Enterobacteriaceae*. Additionally, we tested the models’ ability to classify and distinguish spore forming Gram-positive bacteria, including *B. subtilis*,* B. cereus* and *B. atrophaeus.* Notably, the latter two species are commonly used as simulants for *B. anthracis*, the causative agent of anthrax, in biodefense and counter bioterrorism research programs. Our results demonstrated that eight models achieved perfect performance, with mean accuracy and F1-score of 1.00 ± 0.00 in distinguishing these closely related species (data not shown).

## Cross-validation of ML/DL models with larger external dataset

To determine whether the ML/DL models have truly learned the underlying task rather than memorizing superficial patterns from the training dataset, we conducted a cross-dataset evaluation using spectra from the external RKI MALDI-TOF database. Among the top performing models from the first three tasks, we selected Extra Trees Classifier, Support Vector Classifier and 1-D CNN, to test their ability to predict the bacterial Gram type (binary task) and the bacterial species (multi-class task) based on 285 bacterial spectra obtained from this database.

First, Extra Trees Classifier was chosen as an ensemble ML algorithm that handles non-linearity, it is robust to noise and is commonly applied in high-dimensional data. In contrast, Support Vector Classifier, a kernel ML algorithm, also handles non-linearity, requires feature scaling and is widely used in bioinformatics applications. Finally, we employed 1-D CNN as a DL algorithm which is robust to noise, it handles non-linearity and is applied for signal processing approaches.

### Binary Gram type classification

Table 4 summarizes the classification results for discriminating by bacteria Gram type. The highest performing model is highlighted in bold, and the second-best model is underlined. The evaluation indicates that the Extra Trees Classifier achieved perfect performance with average accuracy and average F1-score of 100%. The 1-D CNN and Support Vector Classifier models also performed well with average accuracy and average F1-score of 97% and 93%, respectively.

**Table 4:** Cross-dataset evaluation using the external dataset for three best-performing ML/DL models in classifying Gram-positive vs. Gram-negative bacteria.

ML/DL Model	Accuracy	F1-score
Extra Trees Classifier	**1.00**	**1.00**
Support Vector Classifier	0.93	0.93
*1-D CNN*	*0.97*	*0.97*

## Multi-class bacterial identification

For the multi-class cross-validation, the results revealed varying levels of generalization across models when tested on unseen data, as shown in Table 5. The Extra Trees Classifier achieved the highest performance, with an average accuracy and average F1-score of 80% on the evaluated bacterial spectra and minimal misclassifications. The 1-D CNN demonstrated slightly lower performance with an average accuracy of 71% and F1-score of 70%. In contrast, the Support Vector Classifier was the least effective, reaching an average accuracy of 54% and average F1-score of 59%.

**Table 5:** Cross-dataset evaluation with the external dataset of three best-performing ML/DL models in classifying bacteria across seven classes.

ML/DL Model	Accuracy	F1-score
Extra Trees Classifier	**0.80**	**0.80**
Support Vector Classifier	0.54	0.59
*1-D CNN*	*0.71*	*0.70*

**Fig. 2:**
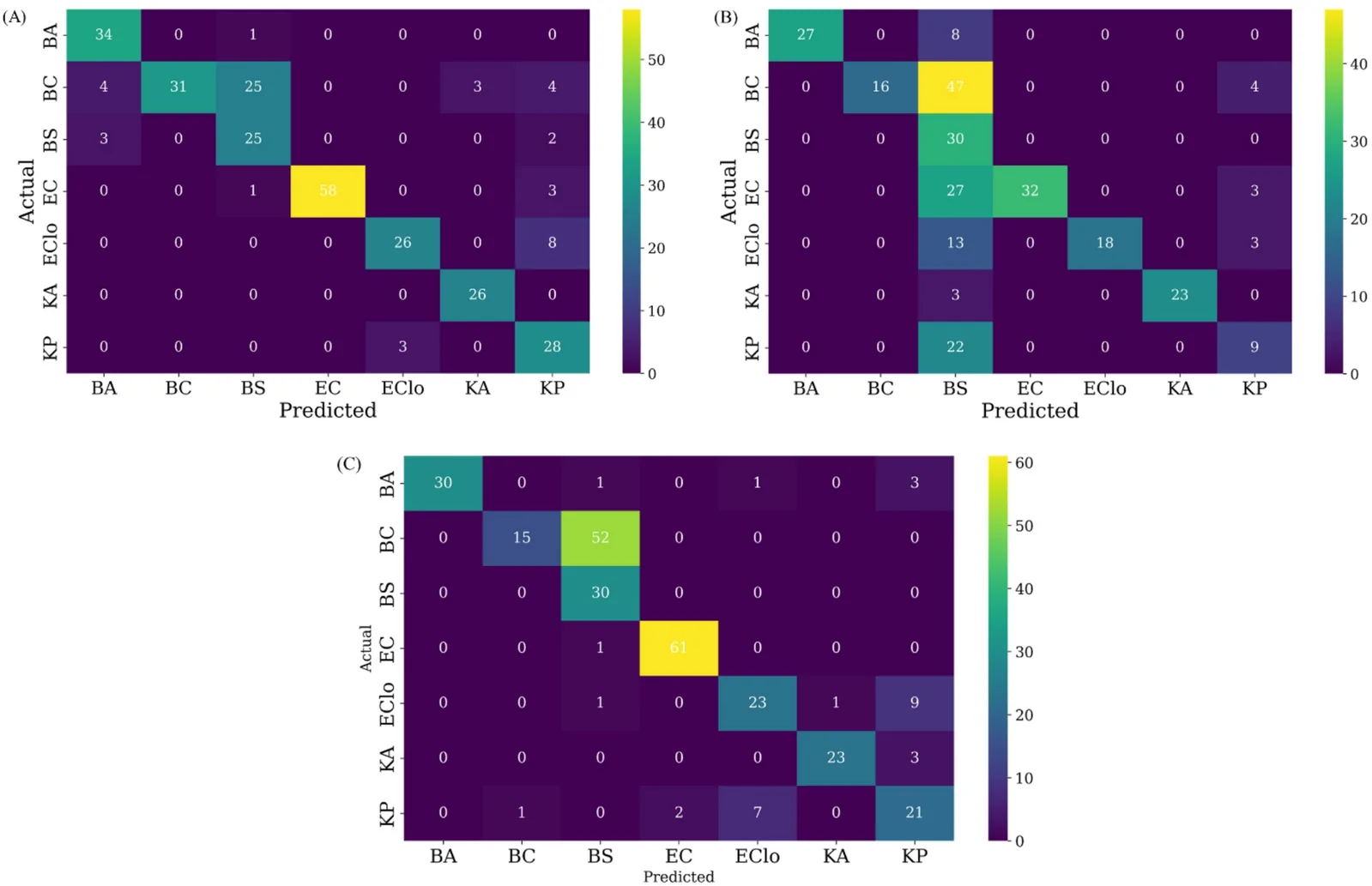
Confusion matrices for three best-performing ML/DL models with the external dataset: (A) Extra Trees Classifier, (B) Support Vector Classifier and (C) 1-D CNN. The bacterial species names in the confusion matrices are presented with abbreviations as follows: BA, *Bacillus atrophaeus*; BC, *Bacillus cereus*; BS, *Bacillus subtilis*; EC, *Escherichia coli*; EClo, *Enterobacter cloacae*; KA, *Klebsiella aerogenes;* and KP, *Klebsiella pneumoniae*.

To further investigate the specific misclassification patterns revealed by the external cross-validation, examining the classification results of mass spectra per class is beneficial. To this end, Fig. 2 presents the confusion matrices of three models evaluated for multi-class classification with mass spectra from the external database. Specifically, the confusion matrix illustrates in the vertical axis the actual classes of the spectra exploited for evaluation, whereas predicted classes are shown in the horizontal axis, meaning that values existing in the diagonal correspond to correct predictions. The results of the evaluation of Extra Trees Classifier are depicted in Fig. 2 (A). For *K. aerogenes*, all spectra (*n* = 26) were correctly predicted. Regarding the other classes included in the external dataset, most of the spectra were correctly classified, except for *B. cereus* where only 31 out of the 67 spectra in total were correctly identified. Afterwards, the classification results of the Support Vector Classifier on the cross-dataset evaluation were investigated. As shown in Fig. 2 (B), only *B. subtilis* was predicted correctly for all spectra (*n* = 30), in comparison to the other 6 classes where a significant number of spectra were misclassified as *B. subtilis*, indicating that the model overfits on the spectra of this specific class. Finally, Fig. 2 (C) presents the results for the 1-D CNN model. All *B. subtilis* spectra were again classified correctly. Additionally, the model succeeded in predicting correctly a significant number of the other 5 classes’ spectra. In particular, pertaining to the *E. coli* class, 61 of 62 spectra were classified correctly. Nevertheless, for *B. cereus*, 52 out of the 67 spectra in total were misclassified as *B. subtilis*, suggesting shared features between these two species within the same genus.

## Discussion

Studies combining MALDI-TOF with AI models to identify and classify viruses remain scarce and challenging in scientific literature. To address this, we developed a comprehensive workflow encompassing viral sample preparation, spectral data acquisition, and model training and evaluation. For this feasibility study, we utilized five viruses representative of various viral groups, including both DNA and RNA viruses, as well as enveloped and non-enveloped viruses. Key steps in this workflow involved viral particle enrichment followed by efficient protein extraction.

In clinical virology, several MALDI-TOF sample preparation approaches have been reported including chemical lysis, solvent-based extraction of viral particles and direct spectral analysis of virus-infected cells compared to an uninfected control^44–46^. The direct analysis method relies on examining spectral changes in the protein profiles of infected cells. This method has been widely used for building viral spectral libraries but often results in spectra contamination by non-specific host-derived proteins. In addition, the method is laborious as it requires culturing viruses and infecting permissive cell lines.

In chemical lysis protocols, detergents, chaotropic agents or solvents are used to extract viral proteins prior to MALDI-TOF MS. These methods may introduce contaminants that can interfere with the ionization process, and their efficiency can be challenged when analyzing low biomass samples. Solvent-based extraction such as acetone precipitation enables selective concentration of viral particles and their proteins. This method also enables sample cleanup from contaminants such as salt, residual detergent and lipids and supports viral protein enrichment. It is particularly relevant for enveloped viruses.

Our protocol integrates an ultrafiltration procedure for virus purification and concentration using 10 kDa filters followed by further solvent-based sample cleanup using acetone precipitation. Our choice of ultrafiltration as the first step was based on our previously published work demonstrating that polyethersulfone membranes (PES) with a 30 kDa cutoff achieved > 4–5 log₁₀ viral retention (> 99.99%) for MS2 and AcNPV^47^. We have adapted this step by using 10 kDa PES filters, which are expected to be at least as efficient as 30 kDa in terms of particle-size exclusion. The subsequent acetone extraction and precipitation of viral particles retained on 10 kDa membranes enables further protein enrichment and removal of residual salts, lipids and contaminants, thereby improving ionization efficiency and producing cleaner spectral signals.

This two-step integrated approach for MALDI-TOF viral sample preparation offers several advantages compared to previously reported methods. It is culture-independent providing a pre-analytical concentration of viral particles present in the sample followed by sample clean up through organic solvent precipitation, which is particularly advantageous for analyzing low-biomass clinical samples.

Collectively, our approach grounded in well-established quantitative filtration performance on viral surrogates and combined with efficient solvent-based extraction practices provides a justified rationale for its enhanced efficiency, robustness and simplicity for viral samples analysis with MALDI-TOF. Applying this sample preparation workflow to viral surrogate samples, followed by data acquisition and model training using 5-fold cross validation procedure, we demonstrated perfect viral classification, with clear distinction from bacterial spectra. Although this approach was applied to a limited number of viruses, it has proven effective, simple, and warrants further evaluation on a broader collection of viral samples.

In the current study, ten AI models were evaluated and demonstrated strong, reliable classification performance across a variety of tasks using our internal MALDI-TOF experimental spectra dataset. Several models achieved high performance in both binary and multi-class classification tasks. Notably, seven models consistently delivered perfect classification results for distinguishing bacteria from viruses, identifying Gram type, and reliably classifying the panel of 12 bacterial and viral samples studied into their respective species, thereby highlighting their precision and robustness. Interestingly, these models showed strong ability to rapidly and accurately differentiate bacteria from viral agents which has direct implications for clinical triage, antimicrobial stewardship, and outbreak management, particularly when rapid decision-making is critical. In addition, the models demonstrate high accuracy in distinguishing closely related species, including *Bacillus* simulants of *B. anthracis*, when assessed on bacterial spectra generated internally underscoring the potential utility of AI-enhanced MALDI-TOF for rapid identification in biodefense scenarios.

To our knowledge, this is among the first studies to demonstrate the feasibility of integrating MALDI-TOF MS with AI models for the classification of both bacterial and viral pathogens, highlighting the potential for broad-spectrum, agent-agnostic detection. The inclusion of viruses in this study, spanning DNA and RNA both enveloped and non-enveloped types, illustrates the adaptability of our workflow to diverse pathogen types, thereby addressing a critical gap in current MALDI-TOF applications.

The usefulness and validity of an AI model depend on its ability to generalize to unseen data, both from external datasets and real-world scenarios. Although our best-performing models Extra Trees Classifier, Support Vector Classifier, and 1-D CNN achieved near perfect performance across various classification tasks on internal datasets, we observed robust accuracy in classifying bacterial species by their Gram type and distinguishing bacterial from viral spectra. However, performance declined notably when cross-validated against spectra from the RKI MALDI-TOF database. This decline was more pronounced with Support Vector Classifier and to a lesser extent with 1-D CNN and Extra Trees Classifier models suggesting potential overfitting. A major and a common source of misclassification was the consistent confusion of *B. cereus* and *B. subtilis* spectra. The persistent misclassification between *B. cereus* and *B. subtilis* can be attributed to the fact that the internal dataset comprised three classes belonging to the *Bacillus* genus, which, in combination with an adequate but not extensive number of spectra for model training, reasonably leads to a non‑negligible rate of misclassification during the evaluation with the external database among species within this genus.

Overall, the performance degradation observed in the cross-dataset evaluation using spectra from the external database occurs because these spectra were generated by different research groups, each employing distinct MALDI-TOF protocols, sample concentrations, and sample preparation procedures. More specifically, trifluoroacetic acid (TFA) was used to inactivate bacteria and prepare samples of the RKI external database, whereas we used direct transfer (DT), extended direct transfer (eDT), and standard protein extraction (PE) recommended by Bruker for producing spectra of our internal dataset. These subtle differences in the extraction protocols may have contributed to the decline of model performance. In addition, these groups applied various instrument calibration standards, further increasing inter-laboratory variability. Consequently, the heterogeneity of the spectra in the external dataset constitutes a critical factor contributing to the reduced classification performance of the models. The observed decrease in performance on the external RKI dataset underscores the challenge of model generalization in MALDI-TOF spectral analysis and highlights the importance of training AI models on heterogeneous and diverse datasets to ensure robustness in real-world applications.

Although MALDI-TOF MS is among the dominant benchmarking methods in the field of microbial identification, the present study is limited by the use of spectra acquired from reference strains under controlled conditions rather than from complex real-world clinical or environmental samples. Sample preparation therefore remains a critical factor, and current workflows still need to move beyond simple direct spotting towards more robust extraction approaches to improve spectral quality in real-world applications. Complex matrices such as blood, urine, or environmental samples can substantially influence MALDI-TOF results by introducing contaminants that suppress analyte ionization, increase background noise, and cause peak interference^48^. As a result, the model performance reported here may not fully capture the variability encountered under routine diagnostic or field conditions.

A further limitation of this study concerns the relatively small size of the dataset, comprising 255 spectra in total, which constrains our ability to draw definitive conclusions regarding the generalizability of the proposed models and increases the risk of overfitting. This consideration is supported by the notably high accuracy and F1-score values observed during model evaluation, although comparable performance has been reported in the literature for analogous classification tasks. Although k-fold cross-validation was implemented to maximize the use of the limited dataset by allocating spectra to both training and validation subsets, this strategy does not fully eliminate the possibility of optimistic performance estimates or overfitting.

Addressing these limitations will require future studies focused on the acquisition of MALDI-TOF spectra from clinical and environmental samples, the use of larger and more heterogeneous datasets, and the systematic evaluation of preprocessing strategies designed to mitigate matrix effects for bacterial and viral pathogens^49,50^. This includes additional scenarios such as aerosol sample collection and accurate bacterial spore discrimination^51,52^, which are particularly relevant for biosecurity applications. Meanwhile, advances in MALDI matrix systems have already demonstrated improved matrix-analyte stoichiometric control^53^ and, together with the rapid development of AI-based tools and expanding databases, provide a strong foundation for the evolution of MALDI-TOF into a high-precision, high-throughput platform in future real-world deployments.

In conclusion, this study involved culturing and preparing bacterial and viral samples to generate an internal MALDI-TOF MS dataset, followed by rigorous spectral pre-processing. Ten models were trained and evaluated for two binary classification tasks and for multi-class classification of seven bacterial species and five viral species. Three top-performing models were further cross-validated using external MALDI-TOF MS spectra from the Robert Koch Institute database. We identified the Extra Trees Classifier and a 1-D CNN as the best-performing approaches for classifying bacterial and viral classes with strong performance metrics within our internal dataset. When these models were assessed and cross-validated against the external MALDI-TOF MS dataset of highly pathogenic bacteria, they demonstrated acceptable performance.

However, further work is required to train these models on broader and more diverse datasets to enhance feature extraction and improve discrimination among closely related species and isolates. The incorporation of a broader range of species and the increase of inter-laboratory data and protocol diversity in datasets reduce the risk of overfitting internal spectral signatures and assists in capturing intra-species variability. Learning curves are considered a reliable tool to investigate the proportion of additional MALDI-TOF spectra required to further improve the performance of trained models. Further work may also include the development of advanced feature extraction methods and deep learning architectures tuned for fine-grained species and strain discrimination.

In addition, we developed a workflow for virus classification that requires evaluation on a larger panel of viral samples to validate its effectiveness, generalizability, and applicability to clinical settings. Overall, these findings illustrate the potential of combining MALDI-TOF MS with AI to create scalable, rapid, and reliable microbial identification platforms for laboratory-based applications. Such platforms could accelerate diagnostics, enhance biodefense preparedness, and provide an adaptable framework for emerging pathogen detection within controlled laboratory environments.

### Methods

The test panel consisted of 12 biological agents including seven bacteria and five viral species (Table 6). The bacterial strains *E. coli (DSM 500)*, *Enterobacter cloacae (DSM109592)*, *K. aerogenes (DSM 30053)*, and *K. pneumoniae* (HUMB 01336), *B. subtilis* (BR 151), *B. atrophaeus* (DSM 675) et *B. cereus (DSM 2302)* were obtained as lyophilized cultures from the German Collection of Microorganisms and Cell cultures (DSMZ) and the Human Microbiome Project culture collection (HUMB; https://eemb.ut.ee). Adeno-associated virus type 2 (AAV2), Moloney murine leukemia virus (MMLV), Lentivirus derived from Human Immunodeficiency Virus 1 (HIV-1) and baculovirus derived from *Autographa californica* multiple nucleopolyhedrovirus (AcMNPV) were purchased from Vectorbuilder Inc (Chicago, IL, USA). Bacteriophage MS2 was propagated in the *E. coli* F + MC-4100/pOX38 host strain using standard cultivation protocol^47^.

**Table 6:** Description of microbial test panel of seven bacteria and five viruses used in the current study. The number of experimental spectra in the internal MALDI-TOF dataset used for training ML/DL models is provided.

Species	Category	No. of MALDI-TOF spectra	Biological features
*Escherichia coli*	Bacteria, Gram**-**	24	Commonly found in the lower intestine of warm-blooded organisms. Most strains are harmless, but some can cause serious food poisoning through production of toxins.
*Enterobacter cloacae*	Bacteria, Gram**-**	22	It is a common member of the gut flora in humans, but it may cause nosocomial infections.
*Klebsiella aerogenes*	Bacteria, Gram**-**	24	A nosocomial, pathogenic bacterium that causes opportunistic infections of most types.
*Klebsiella pneumoniae*	Bacteria, Gram**-**	24	Main cause of hospital-acquired infections and is increasingly becoming resistant to antibiotics. Can cause various infections, including pneumonia, bloodstream infections, urinary tract infections, and wound infections.
*Bacillus subtilis*	Bacteria, Gram**+**	23	Spore forming bacteria that is commonly found in the upper layers of the soil.
*Bacillus atrophaeus*	Bacteria, Gram**+**	24	Black-pigmented spore forming bacteria that is still used as a surrogate for *B. anthracis*, the causative agent of anthrax.
*Bacillus cereus*	Bacteria, Gram**+**	24	Frequently found in soil, food, and marine sponges. It is known to cause toxigenic infections. Certain strains have been identified to carry virulent plasmids like those of *B. anthracis.*
MS2 bacteriophage	Virus, RNA, non-enveloped	17	MS2 is commonly used as an indicator of viruses in wastewater treatment. Its size and morphology are close to that of members of *Picornaviridae*, a family which includes many pathogenic viruses to humans, such as poliovirus.
*Autographa californica multiple nucleopolyhedrovirus (AcMNPV)*	Virus, DNA, enveloped	19	Baculoviruses like *Cydia pomonella granulovirus* and *AcMNPV*, are used as simulants for poxviruses in biodefense research due to their similar physical characteristics and genome structure.
Adeno-associated virus type 2 (AAV2)	Virus, DNA, non-enveloped	17	Virus infecting both dividing and non-dividing cells with low pathogenicity, used in gene transfer studies.
*Moloney murine leukemia virus (MMLV)*	Retrovirus, RNA, enveloped	20	A simple retrovirus widely used for designing gene therapy vectors.
*Human Immunodeficiency Virus 1 (HIV1)*	Retrovirus, RNA, enveloped	17	A retrovirus that specifically targets and destroys CD4 + T cells leading to acquired immunodeficiency syndrome (AIDS).

### Microbial culture and sample preparation

Lyophilized bacterial strains were rehydrated in 500 µL of tryptone soy broth medium (Neogen, USA). A 100 µL aliquot of each suspension was plated on tryptone soy agar (TSA) plates (Neogen, USA) and incubated overnight at 37 °C in 5% CO_2_ atmosphere.

The following day, seven isolated colonies from each TSA plate were selected for protein extraction. Samples were processed using three methods for MALDI-TOF MS analysis recorded by Bruker Microflex MALDI-TOF Mass Spectrometer (Bruker Daltonik, Bremen, Germany): direct transfer (DT), extended direct transfer (eDT) and standard protein extraction (PE), following the manufacturer’s instructions provided in the MALDI Biotyper^®^ protocol (Bruker Daltonics, Billerica, MA, USA). Briefly, in the direct transfer (DT) method, an isolated colony was smeared onto the target MALDI-TOF plate and overlaid with 1 µL of α-cyano-4-hydroxycinnamic acid (HCCA) matrix. In the extended direct transfer (eDT) method, colonies were pretreated with 70% formic acid before HCCA application. The protein extraction was performed according to the Bruker Biotyper^®^ protocol, with mild modifications. Briefly, a sterile 1 µL inoculation loop was used to transfer isolated colonies into 100 µL of HPLC-grade water and mixed thoroughly. Using a pipette, 300 µL of pure ethanol was added to the suspension. After thoroughly mixing, the specimen was centrifuged for two minutes at 14,000 rpm. The supernatant was removed, and the centrifugation step was repeated. Residual ethanol was removed by pipetting. After allowing the pellet to dry at room temperature for a minimum of 5 min, 5 µL of 70% aqueous formic acid was added to re-suspend the pellet, followed by 5 µL of acetonitrile, which was mixed by pipetting. The specimen was then centrifuged for two minutes at 14,000 rpm and 1 µL of supernatant was loaded onto the target plate and overlaid with 1 µL of α-cyano-4-hydroxycinnamic acid (HCCA) matrix.

Viral samples were prepared using centrifugal filter units with a 10 kDa cut-off (Vivaspin^®^ 500, Sartorius, Gottingen, Germany). Briefly, 200 µL of each viral suspension were filtered through the units by centrifugation at 12,000 x g for 10 min. The filtrate was discarded, and the retained viral particles on the filter membrane were washed twice with 400 µL of Milli-Q water, followed by centrifugation under the same conditions. The viral particles were then recovered in 50 µL of Milli-Q water and extracted by thorough mixing with 100 µL of acetone^47^. The samples were heat-inactivated at 70 °C for 10 min and stored at −20 °C until further use. For the MALDI-TOF MS analysis, extracted viral samples were briefly centrifuged and 1 µL of the extract was applied on the target plate, dried at room temperature and overlaid with 1 µL of HCCA matrix.

**Fig. 3:**
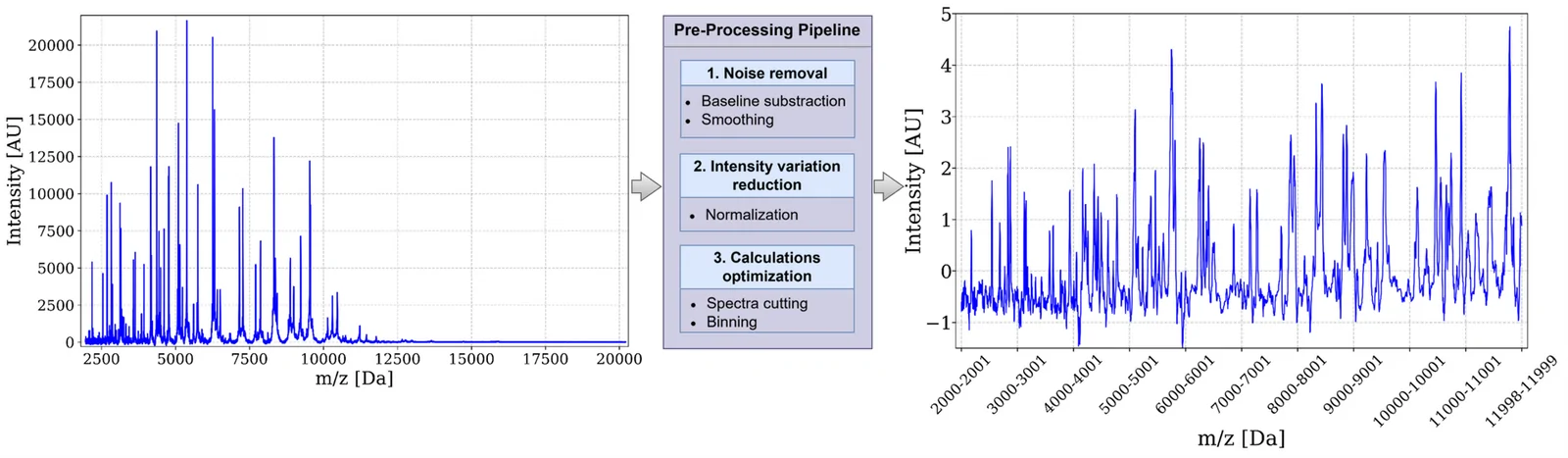
Representative MALDI-TOF spectrum from *E. coli* before (left) and after preprocessing (right).

MALDI generates singly charged ions through pulsed-laser irradiation of the analyte. These ions are subsequently separated into a TOF mass spectrometer based on their velocities prior to detection. The mass-to-charge (m/z) ratios of the ions are determined by measuring the time required for each ion to traverse the flight tube. Figure 3 presents a representative raw mass spectrum obtained from the analysis of *E. coli*. In this plot, the x-axis corresponds to the m/z values of the detected ions, while the y-axis indicates their respective signal intensities. The m/z range spans from 0 to 20,000 Daltons (Da).

### Spectra pre-processing

Raw MALDI-TOF mass spectra were subjected to a standardized pre-processing pipeline using the MicrobeMS software, a MATLAB tool specifically designed by Peter Lasch at the Robert Koch Institute for the analysis of MALDI-TOF mass spectra of microbial samples^54^ as raw data typically contain high levels of noise, baseline drift, and experimental variability that can obscure biologically relevant information and hinder accurate interpretation. The pipeline included baseline subtraction, smoothing, normalization, and spectral cutting. Baseline subtraction was performed using the asymmetric least squares (AsLS) algorithm, which constructs a baseline correction curve through shape-preserving piecewise cubic interpolation over user-defined intervals, effectively removing background noise. Smoothing was applied using the Savitzky-Golay filter to reduce high-frequency noise while preserving peak shape, enhancing signal clarity for downstream analysis. Spectra were then normalized using a modified 1-norm algorithm that computes a scaling factor from intensity differences across selected m/z bins, thereby reducing intensity variation across samples.

Afterwards, spectra were cut to a defined m/z range between 2,000 and 12,000 to remove irrelevant or low-informative regions and reduce memory requirements. All m/z values between 0 and 2,000 Da and the respective intensities were excluded since this region can be crowded with noise and matrix-related peaks which can interfere with the analysis and obscure the signals of interest. Finally, to ensure uniform dimensionality across spectra, intensity measurements were binned based on bin size of 2 Da, resulting in a vector containing 5,000 features that reflects the bins the m/z axis was partitioned. An example of the pre-processed spectrum of *E. coli* is depicted in Fig. 3. All hyperparameters for the pre-processing functions were set to the default values provided by the MicrobeMS software. This pre-processing pipeline ensured consistent spectral quality and improved the reliability of subsequent classification methods in our dataset.

### Classification methods

In this study, we evaluated the performance of various ML and DL models. The ML models included Random Forest, Linear SVC, Ridge Classifier, k-Nearest Neighbors (kNN), Extra Trees Classifier, Support Vector Classifier, Logistic Regression, and XGBoost. Due to their effectiveness in capturing informative patterns within small datasets, we selected these eight ML models for detailed comparison. In addition, two DL baseline methods were also evaluated: a 1-D Convolutional Neural Network (1-D CNN) and a Denoising Autoencoder followed by a Ridge Classifier (DAE-RC). The 1-D CNN was chosen for its ability to extract spatial features from one-dimensional signals, while the DAE-RC was included for its capacity to denoise and reconstruct input data, compressing it into a compact and informative latent representation. Additional details on the models, concise descriptions and representative studies, as well as their respective advantages are summarized in Table 7. The hyperparameters selected for the ML approaches, such as Extra Trees Classifier, SVM, Logistic Regression, Random Forest, kNN, Linear SVC, Ridge Classifier and XGBoost, followed the default recommendations of their respective libraries. In contrast, the DL approaches employed more complex architecture and were fine-tuned prior to the training process. More specifically, the architecture of the CNN-1D model consisted of an initial 1-D convolutional layer with 64 filters and a kernel size of 3 to extract local temporal features from the input sequences, followed by a 1-D max pooling layer with a pool size of 2 to down sample the feature maps and reduce computational complexity. The resulting feature maps were then flattened into a multidimensional vector. Two fully connected Dense layers followed the convolutional block: the first contained 100 units with ReLU activation to capture non-linear relationships, while the final output layer consisted of 10 units, corresponding to 10 classes, with softmax activation for multi-class probability distribution. The model was compiled using categorical cross-entropy loss and the Adam optimizer, with accuracy as the evaluation metric. Regarding the Denoising Autoencoder, it was implemented to reconstruct clean data from noisy inputs while performing dimensionality reduction. The architecture consisted of an input layer accepting 5,000 flattened features, followed by a Gaussian noise layer that corrupted inputs during training to force the model to learn robust features. The encoder phase compressed the data through a dense layer with 256 units with ReLU activation, batch normalization, and 50% dropout regularization to prevent overfitting. A second dense layer further reduced dimensionality to 128 units, forming the bottleneck representation of the decoder phase. Batch normalization was applied to stabilize training and improve gradient flow throughout the network. The model was trained using Mean Squared Error loss to reconstruct original clean data from corrupted inputs, leveraging the combination of Gaussian noise injection and dropout as regularization mechanisms to enhance feature robustness and generalization.

**Table 7:** Detailed descriptions of types, characteristics and representative studies of the ten ML/DL models evaluated in this study. In the “Training Time” column, plus signs indicate the relative time required to train each model, whereas in the “Interpretability” column, asterisks represent the ease of interpreting the model outputs.

Algorithm	Type	Robust to Noise	Training Time	Interpretability	Representative study	Reported performance
**Random Forests**	Ensemble ML	🗹	++	**	Classify *Latilactobacillus sakei* subspecies [35]	Accuracy = 0.95
**Linear Support Vector Classifier**	Linear ML	🗷	+	***	Predict antibiotic resistance in clinically relevant bacteria [38]	Accuracy = 0.82
**Ridge Classifier**	Linear ML	🗹	+	***	Discriminate gut bacteria samples [43]	Accuracy = 1.00
**k Nearest Neighbours**	Instance ML	🗷	+++	**	Discriminate *Enterococcus* species [37]	Accuracy = 0.97
**Extra Trees Classifier**	Ensemble ML	🗹	++	**	Detect microbial forms [41]	Accuracy = 0.97
**Support Vector Classifier**	Kernel ML	🗹	++	**	Classify spectra from *Brucella* species [36]	Accuracy = 1.00
**Logistic Regression**	Linear ML	🗷	+	***	Classify spectra from *Brucella* species [36]	Accuracy = 1.00
**Extreme Gradient Boosting**	Boosting ML	🗹	+	**	Identify *Salmonella* serotype [39]	Accuracy = 0.96
**1-D Convolutional Neural Networks**	ConvNet DL	🗹	++++	*	Detect microbial forms [41]	Accuracy = 0.99
**Denoising Auto Encoder-Ridge Classifier**	Hybrid (DL + Linear ML)	🗹	++++	**	Classify *Listeria* species [42]	Accuracy = 1.00

### Evaluation protocol

**Fig. 4:**
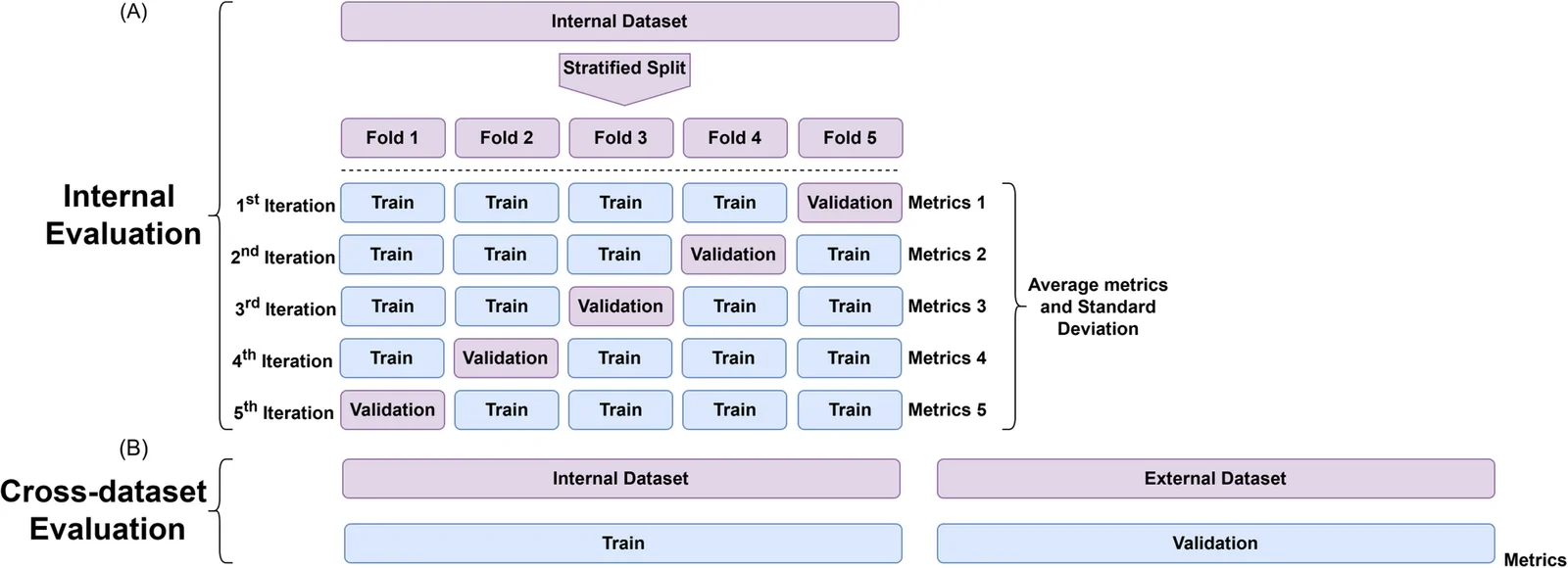
Overview of evaluation protocol applied in ML/DL models using (A) five-fold cross validation for the internal dataset and (B) validation on the total of the external dataset.

Given the fact that each class of the dataset is featured with no more than 24 spectra, a sophisticated method for model evaluation was implemented using 5-fold cross-validation to ensure robust generalization of the relationship between input spectra and target classes. Moreover, 5-fold cross validation substantially mitigates the risk of overfitting by providing a more robust and unbiased estimate of model generalization performance through systematic data partitioning and evaluation. This was achieved by employing a stratified 5-fold split, as illustrated in Fig. 4 (A), wherein 20% of the dataset was allocated for validation in each iteration while maintaining the class distribution. For each experiment and model, training was performed on four folds and validation on the remaining fold, with this process iteratively applied across all five folds.

Classification performance was assessed using the average and standard deviation of accuracy (Eq. 1) and F1-score (Eq. 4) across five iterations, where True Positives (TP), True Negatives (TN), False Positives (FP), and False Negatives (FN) are exploited for the abovementioned evaluation metrics. Regarding accuracy, it represents the proportion of correct predictions (both true positives and true negatives) among the total number of cases examined, whereas F1-score, being the harmonic mean of Precision and Recall, expresses a single metric that reflects how well a classification model identifies positive cases while minimizing both false positives and false negatives. The model that achieves the highest average accuracy with the smallest standard deviation was considered the best-performing model for a given task.

1 $$\:Accuracy=\frac{TP+TN}{TP+TN+FP+FN}$$

2 $$\:Precision=\frac{TP}{TP+FP}$$

3 $$\:Precision=\frac{TP}{TP+FP}$$

4 $$\:F1-score\:=\:2\:\cdot\:\frac{Precision\cdot\:Recall}{Precision+Recall}$$

### Model cross validation methodology

To provide a more realistic assessment of the model’s ability to generalize to new unseen data we validated our ML/DL models with MALDI-TOF mass spectra from an external database^54^. More specifically, spectra from the MALDI-TOF mass spectrometry database of the Robert Koch Institute were selected for further assessment of trained models. This database is freely accessible and can be used without technical restrictions. Concerning the protocols adopted for sample preparation in this database, authors at RKI used TFA as an inactivation protocol for microbial sample preparation, contrary to our work where DT, eDT, and PE were selected for sample preparation. In general, this dataset contains 11,055 spectra from altogether 1,601 bacterial strains and 264 species and is primarily intended to improve the identification and classification of highly pathogenic bacteria. For our experiments, we selected 285 MALDI-TOF spectra for 7 bacteria, 132 for Gram positive bacteria and 153 for Gram negative bacteria, respectively as shown in Table 8. Within the Bacillus genus, 67, 35 and 30 mass spectra were selected for *B. cereus*, *B. atropaheus* and *B. subtilis*, respectively: 62 mass spectra for *E. coli*, 34 for *E. cloacae* and 31 for *K. pneumoniae*. For the second species of the *Klebsiella* genus, *K. aerogenes*, 26 mass spectra were chosen. The information about the bacteria genus, species, strains and id within the database (MicrobeMS ID) of the selected spectra are provided in supplemental table S1 (Table S1). To better explore the selected spectra, more details about the bacteria genus, species, strains and id within the database (MicrobeMS ID) are provided in the supplementary file. The information provided can direct readers to the metadata of the database where description about the NCBI tax id’s, growth time & temperature, growth medium & air, concentration, sample treatment, calibration standard and the measurement method are listed per spectrum. Finally, to perform the cross-dataset validation, we evaluated the performance of three trained models that achieved the highest *accuracy* and F1-score by using the same evaluation metrics after applying the same pre-processing pipeline as applied to the internal dataset. As presented in Fig. 4 (B), the selected models for cross-dataset evaluation were re-trained to the entire bacterial internal dataset prior to their validation to take advantage of all available mass spectra.

**Table 8:** Number of MALDI-TOF mass spectra used from the external dataset to cross-validate trained ML/DL models.

Bacteria Group	Species	No of MALDI-TOF spectra	
Gram positive	*Bacillus cereus*	132	67
	*Bacillus atrophaeus*		35
	*Bacillus subtilis*		30
Gram negative	*Escherichia coli*	153	62
	*Enterobacter cloacae*		34
	*Klebsiella pneumoniae*		31
	*Klebsiella aerogenes*		26

### Software requirements

To conduct the experiments in this study, a laptop with an AMD Ryzen 5 4600 cpu (6 cores, 12 threads) was used with 16 GB of RAM. Initially, spectral preprocessing was performed using the MicrobeMS software package (Microbe MS 0.90d)^54^. Afterwards, Python (3.10) was used for the subsequent stages of our work employing the Pandas (1.4.4) and Pyteomics (5.0) packages for spectra import, scikit-learn (1.1.1) for ML models, XGboost (1.7.3) for gradient boosting, and PyTorch (2.0) for DL models.

## Supplementary Information

Below is the link to the electronic supplementary material.

Supplementary Material 1

## Data Availability

Data are available from the authors upon reasonable request.
