# Supplementary material for: Enhanced classification and identification of bacterial and viral microorganisms by integration of MALDI-TOF mass spectrometry with artificial intelligence
Source: Sci Rep. 2026 Aug 24;16:26407. doi: 10.1038/s41598-026-54426-y (PMC13500668; doi:10.1038/s41598-026-54426-y)
Supplement: Supplementary file 1 [file 41598_2026_54426_MOESM1_ESM.docx]

**Supplementary Data**

**Table S1.** Information about the MALDI-TOF mass spectra used from the external dataset to cross-validate trained ML/DL models.^[1]^

| **Bacteria Group** | **Species** | **No of MALDI-TOF spectra** | | **Spectrum information** | | |
| --- | --- | --- | --- | --- | --- | --- |
|  |  |  |  | *Genus, species, strain* | *MicrobeMS ID* |  |
| *Gram positive* | *Bacillus cereus* | 132 | 67 | Bacillus cereus DSM 2301 | 2022_09_16_Nico_Fischer_0002 |  |
|  |  |  |  | Bacillus cereus DSM 2301 | 2022_09_23_Nico_Fischer_0003 |  |
|  |  |  |  | Bacillus cereus DSM 2301 | 2022_09_23_Nico_Fischer_0002 |  |
|  |  |  |  | Bacillus cereus DSM 2301 | 2022_09_23_Nico_Fischer_0004 |  |
|  |  |  |  | Bacillus cereus DSM 2302 (B192, Beyer) | 2006_16_08_Famulla_Staemmler_0032 |  |
|  |  |  |  | Bacillus cereus DSM 2302 (B192, Beyer) | 2015_04_07_Andy_Schneider_0021 |  |
|  |  |  |  | Bacillus cereus DSM 2302 (B192, Beyer) | 2015_04_07_Andy_Schneider_0023 |  |
|  |  |  |  | Bacillus cereus DSM 2302 (B192, Beyer) | 2015_04_07_Andy_Schneider_0022 |  |
|  |  |  |  | Bacillus cereus DSM 2302 (B192, Beyer) | 2015_04_07_Andy_Schneider_0024 |  |
|  |  |  |  | Bacillus cereus DSM 31 (B181, Beyer) | 2015_03_30_Andy_Schneider_037 |  |
|  |  |  |  | Bacillus cereus DSM 31 (B181, Beyer) | 2015_03_30_Andy_Schneider_039 |  |
|  |  |  |  | Bacillus cereus DSM 31 (B181, Beyer) | 2015_03_30_Andy_Schneider_038 |  |
|  |  |  |  | Bacillus cereus DSM 31 (B181, Beyer) | 2015_03_30_Andy_Schneider_040 |  |
|  |  |  |  | Bacillus cereus A42 | 2021_10_14_Christopher_Hartl_0004 |  |
|  |  |  |  | Bacillus cereus A42 | 2021_10_14_Christopher_Hartl_0003 |  |
|  |  |  |  | Bacillus cereus A42 | 2021_10_14_Christopher_Hartl_0002 |  |
|  |  |  |  | Bacillus cereus A42 | 2021_10_14_Christopher_Hartl_0001 |  |
|  |  |  |  | Bacillus cereus A42 | 2021_10_06_Christopher_Hartl_0004 |  |
|  |  |  |  | Bacillus cereus A42 | 2021_10_06_Christopher_Hartl_0003 |  |
|  |  |  |  | Bacillus cereus A42 | 2021_10_06_Christopher_Hartl_0002 |  |
|  |  |  |  | Bacillus cereus A42 | 2021_10_06_Christopher_Hartl_0001 |  |
|  |  |  |  | Bacillus cereus A31 | 2020_21_10_Charlyn_John_0008 |  |
|  |  |  |  | Bacillus cereus A31 | 2020_21_10_Charlyn_John_0007 |  |
|  |  |  |  | Bacillus cereus A31 | 2020_21_10_Charlyn_John_0006 |  |
|  |  |  |  | Bacillus cereus A31 | 2020_21_10_Charlyn_John_0005 |  |
|  |  |  |  | Bacillus cereus A31 | 2020_21_10_Charlyn_John_0004 |  |
|  |  |  |  | Bacillus cereus A31 | 2020_21_10_Charlyn_John_0003 |  |
|  |  |  |  | Bacillus cereus A31 | 2020_21_10_Charlyn_John_0002 |  |
|  |  |  |  | Bacillus cereus A31 | 2020_21_10_Charlyn_John_0001 |  |
|  |  |  |  | Bacillus cereus A24 | 2020_19_10_Charlyn_John_0008 |  |
|  |  |  |  | Bacillus cereus A24 | 2020_19_10_Charlyn_John_0007 |  |
|  |  |  |  | Bacillus cereus A24 | 2020_19_10_Charlyn_John_0006 |  |
|  |  |  |  | Bacillus cereus A24 | 2020_19_10_Charlyn_John_0005 |  |
|  |  |  |  | Bacillus cereus A24 | 2020_19_10_Charlyn_John_0004 |  |
|  |  |  |  | Bacillus cereus A24 | 2020_19_10_Charlyn_John_0003 |  |
|  |  |  |  | Bacillus cereus A24 | 2020_19_10_Charlyn_John_0002 |  |
|  |  |  |  | Bacillus cereus A24 | 2020_19_10_Charlyn_John_0001 |  |
|  |  |  |  | Bacillus cereus A24 | 2020_28_10_Charlyn_John_0012 |  |
|  |  |  |  | Bacillus cereus A24 | 2020_28_10_Charlyn_John_0011 |  |
|  |  |  |  | Bacillus cereus A24 | 2020_28_10_Charlyn_John_0010 |  |
|  |  |  |  | Bacillus cereus A24 | 2020_28_10_Charlyn_John_0009 |  |
|  |  |  |  | Bacillus cereus A-049 | 2022_04_13_Maren_Staemmler_0004 |  |
|  |  |  |  | Bacillus cereus A-049 | 2022_04_13_Maren_Staemmler_0003 |  |
|  |  |  |  | Bacillus cereus A-049 | 2022_04_13_Maren_Staemmler_0002 |  |
|  |  |  |  | Bacillus cereus A-049 | 2022_04_13_Maren_Staemmler_0001 |  |
|  |  |  |  | Bacillus cereus A-049 | 2022_04_06_Maren_Staemmler_0004 |  |
|  |  |  |  | Bacillus cereus A-049 | 2022_04_06_Maren_Staemmler_0003 |  |
|  |  |  |  | Bacillus cereus A-049 | 2022_04_06_Maren_Staemmler_0002 |  |
|  |  |  |  | Bacillus cereus A-049 | 2022_04_06_Maren_Staemmler_0001 |  |
|  |  |  |  | Bacillus cereus A-049 | 2022_03_31_Maren_Staemmler_0004 |  |
|  |  |  |  | Bacillus cereus A-049 | 2022_03_31_Maren_Staemmler_0003 |  |
|  |  |  |  | Bacillus cereus A-049 | 2022_03_31_Maren_Staemmler_0002 |  |
|  |  |  |  | Bacillus cereus A-049 | 2022_03_31_Maren_Staemmler_0001 |  |
|  |  |  |  | Bacillus cereus 301101RA0432 (RKI) | 2007_23_04_Maren_Staemmler_0009 |  |
|  |  |  |  | Bacillus cereus 301101RA0432 (RKI) | 2007_12_04_Maren_Staemmler_0007 |  |
|  |  |  |  | Bacillus cereus 2617 | 2022_04_13_Maren_Staemmler_0014 |  |
|  |  |  |  | Bacillus cereus 2617 | 2022_04_13_Maren_Staemmler_0016 |  |
|  |  |  |  | Bacillus cereus 2617 | 2022_04_13_Maren_Staemmler_0013 |  |
|  |  |  |  | Bacillus cereus 2617 | 2022_04_06_Maren_Staemmler_0016 |  |
|  |  |  |  | Bacillus cereus 2617 | 2022_04_06_Maren_Staemmler_0015 |  |
|  |  |  |  | Bacillus cereus 2617 | 2022_04_06_Maren_Staemmler_0014 |  |
|  |  |  |  | Bacillus cereus 2617 | 2022_04_06_Maren_Staemmler_0013 |  |
|  |  |  |  | Bacillus cereus unknown origin (B33, Beyer) | 2006_11_08_Famulla_Staemmler_0031 |  |
|  |  |  |  | Bacillus cereus unknown origin (B33, Beyer) | 2014_12_10_Andy_Schneider_0009 |  |
|  |  |  |  | Bacillus cereus unknown origin (B33, Beyer) | 2014_12_10_Andy_Schneider_0011 |  |
|  |  |  |  | Bacillus cereus unknown origin (B33, Beyer) | 2014_12_10_Andy_Schneider_0010 |  |
|  |  |  |  | Bacillus cereus unknown origin (B33, Beyer) | 2014_12_10_Andy_Schneider_0012 |  |
|  | *Bacillus atrophaeus* |  | 35 | Bacillus atrophaeus ABI02A | 2017_03_13_Julia_Mitschke_0021 |  |
|  |  |  |  | Bacillus atrophaeus ABI02A | 2017_03_13_Julia_Mitschke_0023 |  |
|  |  |  |  | Bacillus atrophaeus ABI02A | 2017_03_13_Julia_Mitschke_0022 |  |
|  |  |  |  | Bacillus atrophaeus ABI02A | 2017_03_13_Julia_Mitschke_0024 |  |
|  |  |  |  | Bacillus atrophaeus ABI02A | 2017_07_14_Julia_Mitschke_0013 |  |
|  |  |  |  | Bacillus atrophaeus ABI02A | 2017_07_14_Julia_Mitschke_0015 |  |
|  |  |  |  | Bacillus atrophaeus ABI02A | 2017_07_14_Julia_Mitschke_0014 |  |
|  |  |  |  | Bacillus atrophaeus ABI02A | 2017_07_14_Julia_Mitschke_0016 |  |
|  |  |  |  | Bacillus atrophaeus ABI02A | 2018_01_11_Julia_Mitschke_0013 |  |
|  |  |  |  | Bacillus atrophaeus ABI02A | 2018_01_11_Julia_Mitschke_0015 |  |
|  |  |  |  | Bacillus atrophaeus ABI02A | 2018_01_11_Julia_Mitschke_0014 |  |
|  |  |  |  | Bacillus atrophaeus ABI02A | 2018_01_11_Julia_Mitschke_0016 |  |
|  |  |  |  | Bacillus atrophaeus ABI05A | 2017_03_13_Julia_Mitschke_0025 |  |
|  |  |  |  | Bacillus atrophaeus ABI05A | 2017_03_13_Julia_Mitschke_0027 |  |
|  |  |  |  | Bacillus atrophaeus ABI05A | 2017_03_13_Julia_Mitschke_0026 |  |
|  |  |  |  | Bacillus atrophaeus ABI05A | 2017_03_13_Julia_Mitschke_0025 |  |
|  |  |  |  | Bacillus atrophaeus ABI05A | 2017_03_13_Julia_Mitschke_0028 |  |
|  |  |  |  | Bacillus atrophaeus ABI05A | 2017_07_14_Julia_Mitschke_0019 |  |
|  |  |  |  | Bacillus atrophaeus ABI05A | 2017_07_14_Julia_Mitschke_0017 |  |
|  |  |  |  | Bacillus atrophaeus ABI05A | 2017_07_14_Julia_Mitschke_0020 |  |
|  |  |  |  | Bacillus atrophaeus ABI05A | 2017_07_14_Julia_Mitschke_0018 |  |
|  |  |  |  | Bacillus atrophaeus ABI05A | 2018_01_11_Julia_Mitschke_0005 |  |
|  |  |  |  | Bacillus atrophaeus ABI05A | 2018_01_11_Julia_Mitschke_0007 |  |
|  |  |  |  | Bacillus atrophaeus ABI05A | 2018_01_11_Julia_Mitschke_0006 |  |
|  |  |  |  | Bacillus atrophaeus ABI05A | 2018_01_11_Julia_Mitschke_0008 |  |
|  |  |  |  | Bacillus atrophaeus ALEQ 1 | 2022_05_24_Nico_Fischer_0013 |  |
|  |  |  |  | Bacillus atrophaeus ALEQ 1 | 2022_05_24_Nico_Fischer_0015 |  |
|  |  |  |  | Bacillus atrophaeus ALEQ 1 | 2022_05_24_Nico_Fischer_0014 |  |
|  |  |  |  | Bacillus atrophaeus ALEQ 1 | 2022_05_24_Nico_Fischer_0016 |  |
|  |  |  |  | Bacillus atrophaeus ALEQ 1 | 2022_05_24_Nico_Fischer_0033 |  |
|  |  |  |  | Bacillus atrophaeus ALEQ 1 | 2022_05_24_Nico_Fischer_0035 |  |
|  |  |  |  | Bacillus atrophaeus ALEQ 1 | 2022_05_24_Nico_Fischer_0034 |  |
|  |  |  |  | Bacillus atrophaeus ALEQ 1 | 2022_05_24_Nico_Fischer_0036 |  |
|  |  |  |  | Bacillus atrophaeus ALEQ 1 | 2022_06_14_Nico_Fischer_0001 |  |
|  |  |  |  | Bacillus atrophaeus ALEQ 1 | 2022_06_14_Nico_Fischer_0003 |  |
|  | *Bacillus subtilis* |  | 30 | Bacillus spizizenii DSM 347 ATCC 6633 (B13, Beyer) | 2006_11_08_Famulla_Staemmler_0014 |  |
|  |  |  |  | Bacillus subtilis unknown origin (B12, Beyer) | 2006_11_08_Famulla_Staemmler_0012 |  |
|  |  |  |  | Bacillus spizizenii glaxo (B130, Beyer) | 2006_15_08_Famulla_Staemmler_0031 |  |
|  |  |  |  | Bacillus subtilis glaxo (B129, Beyer) | 2006_15_08_Famulla_Staemmler_0030 |  |
|  |  |  |  | Bacillus subtilis unknown origin (B20, Beyer) | 2006_11_08_Famulla_Staemmler_0020 |  |
|  |  |  |  | Bacillus spizizenii unknown origin (B1, Beyer) | 2014_12_01_Andy_Schneider_0004 |  |
|  |  |  |  | Bacillus spizizenii unknown origin (B1, Beyer) | 2014_12_01_Andy_Schneider_0002 |  |
|  |  |  |  | Bacillus spizizenii unknown origin (B1, Beyer) | 2014_12_01_Andy_Schneider_0001 |  |
|  |  |  |  | Bacillus spizizenii unknown origin (B1, Beyer) | 2014_12_01_Andy_Schneider_0003 |  |
|  |  |  |  | Bacillus spizizenii glaxo (B130, Beyer) | 2015_03_27_Andy_Schneider_0005 |  |
|  |  |  |  | Bacillus spizizenii glaxo (B130, Beyer) | 2015_03_27_Andy_Schneider_0006 |  |
|  |  |  |  | Bacillus spizizenii glaxo (B130, Beyer) | 2015_03_27_Andy_Schneider_0007 |  |
|  |  |  |  | Bacillus spizizenii glaxo (B130, Beyer) | 2015_03_27_Andy_Schneider_0008 |  |
|  |  |  |  | Bacillus spizizenii DSM 347 ATCC 6633 (B13, Beyer) | 2014_12_01_Andy_Schneider_0037 |  |
|  |  |  |  | Bacillus spizizenii DSM 347 ATCC 6633 (B13, Beyer) | 2014_12_01_Andy_Schneider_0038 |  |
|  |  |  |  | Bacillus spizizenii DSM 347 ATCC 6633 (B13, Beyer) | 2014_12_01_Andy_Schneider_0039 |  |
|  |  |  |  | Bacillus spizizenii DSM 347 ATCC 6633 (B13, Beyer) | 2014_12_01_Andy_Schneider_0040 |  |
|  |  |  |  | Bacillus sp. unknown origin (B14, Beyer) | 2014_12_02_Andy_Schneider_0001 |  |
|  |  |  |  | Bacillus sp. unknown origin (B14, Beyer) | 2014_12_02_Andy_Schneider_0002 |  |
|  |  |  |  | Bacillus sp. unknown origin (B14, Beyer) | 2014_12_02_Andy_Schneider_0003 |  |
|  |  |  |  | Bacillus subtilis unknown origin (B12, Beyer) | 2014_12_01_Andy_Schneider_0033 |  |
|  |  |  |  | Bacillus subtilis unknown origin (B12, Beyer) | 2014_12_01_Andy_Schneider_0034 |  |
|  |  |  |  | Bacillus subtilis unknown origin (B12, Beyer) | 2014_12_01_Andy_Schneider_0035 |  |
|  |  |  |  | Bacillus subtilis unknown origin (B12, Beyer) | 2014_12_01_Andy_Schneider_0036 |  |
|  |  |  |  | Bacillus subtilis glaxo (B129, Beyer) | 2015_03_27_Andy_Schneider_0001 |  |
|  |  |  |  | Bacillus subtilis glaxo (B129, Beyer) | 2015_03_27_Andy_Schneider_0002 |  |
|  |  |  |  | Bacillus subtilis glaxo (B129, Beyer) | 2015_03_27_Andy_Schneider_0003 |  |
|  |  |  |  | Bacillus subtilis glaxo (B129, Beyer) | 2015_03_27_Andy_Schneider_0004 |  |
|  |  |  |  | Bacillus subtilis B rauh (RKI) | 2007_23_04_Maren_Staemmler_0012 |  |
|  |  |  |  | Bacillus subtilis B rauh (RKI) | 2007_23_04_Maren_Staemmler_0008 |  |
| *Gram negative* | *Escherichia coli* | 153 | 62 | Escherichia coli Nm I | 2008_09_06_Maren_Staemmler_0006 |  |
|  |  |  |  | Escherichia coli Nm I | 2008_09_05_Maren_Staemmler_0006 |  |
|  |  |  |  | Escherichia coli 08\|01585 | 2008_09_06_Maren_Staemmler_0007 |  |
|  |  |  |  | Escherichia coli 08\|01585 | 2008_09_05_Maren_Staemmler_0007 |  |
|  |  |  |  | Escherichia coli A-002-43 Le | 2017_05_01_Andy_Schneider_0013 |  |
|  |  |  |  | Escherichia coli A-002-43 Le | 2017_05_01_Andy_Schneider_0014 |  |
|  |  |  |  | Escherichia coli A-002-43 Le | 2017_05_01_Andy_Schneider_0015 |  |
|  |  |  |  | Escherichia coli A-002-43 Le | 2017_05_01_Andy_Schneider_0016 |  |
|  |  |  |  | Escherichia coli A-002-43 Le | 2017_05_30_Andy_Schneider_0007 |  |
|  |  |  |  | Escherichia coli A-002-43 Le | 2017_05_30_Andy_Schneider_0008 |  |
|  |  |  |  | Escherichia coli Ring trial A-269_07 B | 2012_04_02_Maren_Staemmler_0014 |  |
|  |  |  |  | Escherichia coli Ring trial A-269_07 B | 2012_04_04_Maren_Staemmler_0014 |  |
|  |  |  |  | Escherichia coli DSM 30083(T) | 2021_12_02_Christopher_Hartl_0001 |  |
|  |  |  |  | Escherichia coli DSM 30083(T) | 2021_12_02_Christopher_Hartl_0002 |  |
|  |  |  |  | Escherichia coli DSM 30083(T) | 2021_12_02_Christopher_Hartl_0003 |  |
|  |  |  |  | Escherichia coli DSM 30083(T) | 2021_12_02_Christopher_Hartl_0004 |  |
|  |  |  |  | Escherichia coli DSM 30083(T) | 2021_12_03_Anne_Gries_0001 |  |
|  |  |  |  | Escherichia coli DSM 30083(T) | 2021_12_03_Anne_Gries_0002 |  |
|  |  |  |  | Escherichia coli DSM 30083(T) | 2021_12_03_Anne_Gries_0003 |  |
|  |  |  |  | Escherichia coli DSM 30083(T) | 2021_12_03_Anne_Gries_0004 |  |
|  |  |  |  | Escherichia coli DSM 30083(T) | 2021_12_08_Anne_Gries_0001 |  |
|  |  |  |  | Escherichia coli DSM 30083(T) | 2021_12_08_Anne_Gries_0002 |  |
|  |  |  |  | Escherichia coli DSM 30083(T) | 2021_12_08_Anne_Gries_0003 |  |
|  |  |  |  | Escherichia coli DSM 30083(T) | 2021_12_08_Anne_Gries_0004 |  |
|  |  |  |  | Escherichia coli DSM 30083(T) | 2022_03_18_Anne_Gries_0009 |  |
|  |  |  |  | Escherichia coli DSM 30083(T) | 2022_03_18_Anne_Gries_0010 |  |
|  |  |  |  | Escherichia coli DSM 30083(T) | 2022_03_18_Anne_Gries_0011 |  |
|  |  |  |  | Escherichia coli DSM 30083(T) | 2022_03_18_Anne_Gries_0012 |  |
|  |  |  |  | Escherichia coli DSM 3871 | 2016_06_27_Andy_Schneider_0001 |  |
|  |  |  |  | Escherichia coli DSM 3871 | 2016_06_27_Andy_Schneider_0002 |  |
|  |  |  |  | Escherichia coli DSM 3871 | 2016_06_27_Andy_Schneider_0003 |  |
|  |  |  |  | Escherichia coli DSM 3871 | 2016_06_27_Andy_Schneider_0004 |  |
|  |  |  |  | Escherichia coli DSM 3871 | 2016_06_10_Andy_Schneider_0001 |  |
|  |  |  |  | Escherichia coli DSM 3871 | 2016_06_10_Andy_Schneider_0002 |  |
|  |  |  |  | Escherichia coli DSM 3871 | 2016_06_10_Andy_Schneider_0003 |  |
|  |  |  |  | Escherichia coli DSM 3871 | 2016_06_10_Andy_Schneider_0004 |  |
|  |  |  |  | Escherichia coli DSM 3871 | 2016_06_10_Andy_Schneider_0005 |  |
|  |  |  |  | Escherichia coli DSM 3871 | 2016_06_10_Andy_Schneider_0006 |  |
|  |  |  |  | Escherichia coli DSM 3871 | 2008_18_04_Maren_Staemmler_0009 |  |
|  |  |  |  | Escherichia coli DSM 3871 | 2008_04_11_Maren_Staemmler_0009 |  |
|  |  |  |  | Escherichia coli DSM 3871 | 2016_07_04_Andy_Schneider_0009 |  |
|  |  |  |  | Escherichia coli DSM 3871 | 2016_07_04_Andy_Schneider_0010 |  |
|  |  |  |  | Escherichia coli DSM 3871 | 2016_07_04_Andy_Schneider_0011 |  |
|  |  |  |  | Escherichia coli DSM 3871 | 2016_07_04_Andy_Schneider_0012 |  |
|  |  |  |  | Escherichia coli DSM 3871 | 2016_06_27_Andy_Schneider_0010 |  |
|  |  |  |  | Escherichia coli DSM 3871 | 2016_06_27_Andy_Schneider_0011 |  |
|  |  |  |  | Escherichia coli DSM 3871 | 2016_06_27_Andy_Schneider_0012 |  |
|  |  |  |  | Escherichia coli DSM 3871 | 2016_06_27_Andy_Schneider_0013 |  |
|  |  |  |  | Escherichia coli NCTC 10418, DSM 682, ATCC 10536, NCIB 8879 | 2008_09_05_Maren_Staemmler_0008 |  |
|  |  |  |  | Escherichia coli NCTC 10418, DSM 682, ATCC 10536, NCIB 8879 | 2008_09_06_Maren_Staemmler_0008 |  |
|  |  |  |  | Escherichia coli RKI A139 | 2008_16_05_Maren_Staemmler_0003 |  |
|  |  |  |  | Escherichia coli RKI A139 | 2008_04_04_Maren_Staemmler_0003 |  |
|  |  |  |  | Escherichia coli RKI A139 | 2008_02_04_Maren_Staemmler_0003 |  |
|  |  |  |  | Escherichia coli RKI A139 | 2008_04_09_Maren_Staemmler_0003 |  |
|  |  |  |  | Escherichia coli RKI A139 | 2008_18_04_Maren_Staemmler_0010 |  |
|  |  |  |  | Escherichia coli RKI A139 | 2008_04_11_Maren_Staemmler_0010 |  |
|  |  |  |  | Escherichia coli RKI A139 | 2008_25_04_Maren_Staemmler_0003 |  |
|  |  |  |  | Escherichia coli RKI A139 | 2008_20_02_Maren_Staemmler_0003 |  |
|  |  |  |  | Escherichia coli RKI A139 | 2012_08_17_Andy_Schneider_0005 |  |
|  |  |  |  | Escherichia coli RKI A139 | 2012_08_17_Andy_Schneider_0011 |  |
|  |  |  |  | Escherichia coli RKI A139 | 2012_08_17_Andy_Schneider_0002 |  |
|  |  |  |  | Escherichia coli RKI A139 | 2012_08_17_Andy_Schneider_0008 |  |
|  | *Enterobacter cloacae* |  | 34 | Enterobacter cloacae B29 | 2011_10_14_Stefanie_Herfort_0023 |  |
|  |  |  |  | Enterobacter cloacae B29 | 2012_01_10_Stefanie_Herfort_0023 |  |
|  |  |  |  | Enterobacter cloacae B29 | 2012_01_26_Stefanie_Herfort_0013 |  |
|  |  |  |  | Enterobacter cloacae B824 | 2011_10_14_Stefanie_Herfort_0025 |  |
|  |  |  |  | Enterobacter cloacae B824 | 2012_01_10_Stefanie_Herfort_0025 |  |
|  |  |  |  | Enterobacter cloacae B824 | 2012_01_26_Stefanie_Herfort_0015 |  |
|  |  |  |  | Enterobacter cloacae B861 | 2011_10_14_Stefanie_Herfort_0024 |  |
|  |  |  |  | Enterobacter cloacae B861 | 2012_01_10_Stefanie_Herfort_0024 |  |
|  |  |  |  | Enterobacter cloacae B861 | 2012_01_26_Stefanie_Herfort_0014 |  |
|  |  |  |  | Enterobacter cloacae B9 | 2011_10_14_Stefanie_Herfort_0022 |  |
|  |  |  |  | Enterobacter cloacae B9 | 2012_01_10_Stefanie_Herfort_0022 |  |
|  |  |  |  | Enterobacter cloacae B9 | 2012_01_26_Stefanie_Herfort_0012 |  |
|  |  |  |  | Enterobacter cloacae E118 | 2011_10_14_Stefanie_Herfort_0013 |  |
|  |  |  |  | Enterobacter cloacae E118 | 2012_01_10_Stefanie_Herfort_0013 |  |
|  |  |  |  | Enterobacter cloacae E118 | 2012_01_26_Stefanie_Herfort_0003 |  |
|  |  |  |  | Enterobacter cloacae E15 | 2011_11_10_Stefanie_Herfort_0004 |  |
|  |  |  |  | Enterobacter cloacae E15 | 2012_01_09_Stefanie_Herfort_0004 |  |
|  |  |  |  | Enterobacter cloacae E15 | 2011_11_17_Stefanie_Herfort_0004 |  |
|  |  |  |  | Enterobacter cloacae E151 | 2011_10_14_Stefanie_Herfort_0014 |  |
|  |  |  |  | Enterobacter cloacae E151 | 2012_01_10_Stefanie_Herfort_0014 |  |
|  |  |  |  | Enterobacter cloacae E151 | 2012_01_26_Stefanie_Herfort_0004 |  |
|  |  |  |  | Enterobacter cloacae E34 | 2012_01_09_Stefanie_Herfort_0006 |  |
|  |  |  |  | Enterobacter cloacae E34 | 2011_11_10_Stefanie_Herfort_0006 |  |
|  |  |  |  | Enterobacter cloacae E34 | 2011_11_17_Stefanie_Herfort_0006 |  |
|  |  |  |  | Enterobacter cloacae E36 | 2012_01_09_Stefanie_Herfort_0007 |  |
|  |  |  |  | Enterobacter cloacae E36 | 2011_11_10_Stefanie_Herfort_0007 |  |
|  |  |  |  | Enterobacter cloacae E36 | 2011_11_17_Stefanie_Herfort_0007 |  |
|  |  |  |  | Enterobacter cloacae E46 | 2011_11_10_Stefanie_Herfort_0008 |  |
|  |  |  |  | Enterobacter cloacae E46 | 2012_01_09_Stefanie_Herfort_0008 |  |
|  |  |  |  | Enterobacter cloacae E46 | 2011_11_17_Stefanie_Herfort_0008 |  |
|  |  |  |  | Enterobacter cloacae ssp. cloacae DSM 30054, ATCC 1304, NCTC 10005 | 2008_18_04_Maren_Staemmler_0007 |  |
|  |  |  |  | Enterobacter cloacae ssp. cloacae DSM 30054, ATCC 1304, NCTC 10005 | 2008_04_11_Maren_Staemmler_0007 |  |
|  |  |  |  | Enterobacter cloacae ssp. cloacae DSM 30054, ATCC 1304, NCTC 10005 | 2008_09_06_Maren_Staemmler_0003 |  |
|  |  |  |  | Enterobacter cloacae ssp. cloacae DSM 30054, ATCC 1304, NCTC 1000 | 2008_09_05_Maren_Staemmler_0003 |  |
|  | *Klebsiella pneumoniae* |  | 31 | Klebsiella pneumoniae DSM 681, ATCC 10031, NCTC 7427 | 2008_04_11_Maren_Staemmler_0026 |  |
|  |  |  |  | Klebsiella pneumoniae DSM 681, ATCC 10031, NCTC 7427 | 2008_21_04_Maren_Staemmler_0002 |  |
|  |  |  |  | Klebsiella pneumoniae ssp. ozaenae ATCC 25926, NCDC 2827-70 | 2008_18_04_Maren_Staemmler_0012 |  |
|  |  |  |  | Klebsiella pneumoniae ssp. ozaenae ATCC 25926, NCDC 2827-70 | 2008_04_11_Maren_Staemmler_0012 |  |
|  |  |  |  | Klebsiella pneumoniae ssp. ozaenae ATCC 25926, NCDC 2827-70 | 2013_11_01_Maren_Staemmler_0017 |  |
|  |  |  |  | Klebsiella pneumoniae ssp. ozaenae ATCC 25926, NCDC 2827-70 | 2013_11_11_Maren_Staemmler_0017 |  |
|  |  |  |  | Klebsiella pneumoniae ssp. ozaenae ATCC 25926, NCDC 2827-70 | 2013_10_07_Maren_Staemmler_0017 |  |
|  |  |  |  | Klebsiella pneumoniae ssp. pneumoniae DSM 30104 | 2022_05_27_Anne_Gries_0021 |  |
|  |  |  |  | Klebsiella pneumoniae ssp. pneumoniae DSM 30104 | 2022_05_27_Anne_Gries_0022 |  |
|  |  |  |  | Klebsiella pneumoniae ssp. pneumoniae DSM 30104 | 2022_05_27_Anne_Gries_0023 |  |
|  |  |  |  | Klebsiella pneumoniae ssp. pneumoniae DSM 30104 | 2022_05_27_Anne_Gries_0024 |  |
|  |  |  |  | Klebsiella quasipneumoniae ssp. quasipneumoniae DSM 28211 | 2022_05_27_Anne_Gries_0025 |  |
|  |  |  |  | Klebsiella quasipneumoniae ssp. quasipneumoniae DSM 28211 | 2022_05_27_Anne_Gries_0026 |  |
|  |  |  |  | Klebsiella quasipneumoniae ssp. quasipneumoniae DSM 28211 | 2022_05_27_Anne_Gries_0027 |  |
|  |  |  |  | Klebsiella quasipneumoniae ssp. quasipneumoniae DSM 28211 | 2022_05_27_Anne_Gries_0028 |  |
|  |  |  |  | Klebsiella quasipneumoniae ssp. similipneumoniae DSM 28212 | 2022_05_27_Anne_Gries_0029 |  |
|  |  |  |  | Klebsiella quasipneumoniae ssp. similipneumoniae DSM 28212 | 2022_05_27_Anne_Gries_0030 |  |
|  |  |  |  | Klebsiella quasipneumoniae ssp. similipneumoniae DSM 28212 | 2022_05_27_Anne_Gries_0031 |  |
|  |  |  |  | Klebsiella quasipneumoniae ssp. similipneumoniae DSM 28212 | 2022_05_27_Anne_Gries_0032 |  |
|  |  |  |  | Klebsiella oxytoca ATCC 13182, DSM 5175 | 2013_11_01_Maren_Staemmler_0016 |  |
|  |  |  |  | Klebsiella oxytoca ATCC 13182, DSM 5175 | 2013_11_11_Maren_Staemmler_0016 |  |
|  |  |  |  | Klebsiella oxytoca ATCC 13182, DSM 5175 | 2013_10_07_Maren_Staemmler_0016 |  |
|  |  |  |  | Klebsiella oxytoca ATCC 13182, DSM 5175 | 2008_04_11_Maren_Staemmler_0025 |  |
|  |  |  |  | Klebsiella oxytoca ATCC 13182, DSM 5175 | 2008_21_04_Maren_Staemmler_0001 |  |
|  |  |  |  | Klebsiella oxytoca CB4063 | 2013_11_01_Maren_Staemmler_0011 |  |
|  |  |  |  | Klebsiella oxytoca CB4063 | 2013_11_11_Maren_Staemmler_0011 |  |
|  |  |  |  | Klebsiella oxytoca CB4063 | 2013_10_07_Maren_Staemmler_0011 |  |
|  |  |  |  | Klebsiella oxytoca CB4072 | 2013_11_01_Maren_Staemmler_0012 |  |
|  |  |  |  | Klebsiella oxytoca CB4072 | 2013_11_11_Maren_Staemmler_0012 |  |
|  |  |  |  | Klebsiella oxytoca CB4072 | 2013_10_07_Maren_Staemmler_0012 |  |
|  |  |  |  | Klebsiella oxytoca CB4074 | 2013_11_01_Maren_Staemmler_0013 |  |
|  | *Klebsiella aerogenes* |  | 26 | Klebsiella aerogenes DSM 30053, ATCC 13048, DSM 26516, NCTC 10006, LMG 2094, CCUG 1429 | 2008_18_04_Maren_Staemmler_0005 |  |
|  |  |  |  | Klebsiella aerogenes DSM 30053, ATCC 13048, DSM 26516, NCTC 10006, LMG 2094, CCUG 1429 | 2008_04_11_Maren_Staemmler_0005 |  |
|  |  |  |  | Klebsiella aerogenes DSM 30104 | 2022_05_27_Anne_Gries_0018 |  |
|  |  |  |  | Klebsiella aerogenes DSM 30053 | 2022_05_27_Anne_Gries_0017 |  |
|  |  |  |  | Klebsiella aerogenes DSM 30053 | 2022_05_27_Anne_Gries_0020 |  |
|  |  |  |  | Klebsiella aerogenes DSM 28211 | 2022_05_27_Anne_Gries_0019 |  |
|  |  |  |  | Klebsiella aerogenes E111 | 2011_10_14_Stefanie_Herfort_0011 |  |
|  |  |  |  | Klebsiella aerogenes E111 | 2012_01_10_Stefanie_Herfort_0011 |  |
|  |  |  |  | Klebsiella aerogenes E111 | 2012_01_26_Stefanie_Herfort_0001 |  |
|  |  |  |  | Klebsiella aerogenes E115 | 2011_10_14_Stefanie_Herfort_0012 |  |
|  |  |  |  | Klebsiella aerogenes E115 | 2012_01_10_Stefanie_Herfort_0012 |  |
|  |  |  |  | Klebsiella aerogenes E115 | 2012_01_26_Stefanie_Herfort_0002 |  |
|  |  |  |  | Klebsiella aerogenes E161 | 2011_10_14_Stefanie_Herfort_0015 |  |
|  |  |  |  | Klebsiella aerogenes E161 | 2012_01_10_Stefanie_Herfort_0015 |  |
|  |  |  |  | Klebsiella aerogenes E161 | 2012_01_26_Stefanie_Herfort_0005 |  |
|  |  |  |  | Klebsiella aerogenes E19 | 2012_01_09_Stefanie_Herfort_0005 |  |
|  |  |  |  | Klebsiella aerogenes E19 | 2011_11_10_Stefanie_Herfort_0005 |  |
|  |  |  |  | Klebsiella aerogenes E19 | 2011_11_17_Stefanie_Herfort_0005 |  |
|  |  |  |  | Klebsiella aerogenes E56 | 2011_11_10_Stefanie_Herfort_0009 |  |
|  |  |  |  | Klebsiella aerogenes E56 | 2012_01_09_Stefanie_Herfort_0009 |  |
|  |  |  |  | Klebsiella aerogenes E56 | 2011_11_17_Stefanie_Herfort_0009 |  |
|  |  |  |  | Klebsiella aerogenes E69 | 2011_11_10_Stefanie_Herfort_0010 |  |
|  |  |  |  | Klebsiella aerogenes R69 | 2012_01_09_Stefanie_Herfort_0010 |  |
|  |  |  |  | Klebsiella aerogenes R69 | 2011_11_17_Stefanie_Herfort_0010 |  |
|  |  |  |  | Klebsiella aerogenes NM 20 | 2008_09_06_Maren_Staemmler_0005 |  |
|  |  |  |  | Klebsiella aerogenes NM 20 | 2008_09_05_Maren_Staemmler_0005 |  |

1. [Version 4.2 (20230306) of the MALDI-ToF Mass Spectrometry Database for Identification and Classification of Highly Pathogenic Microorganisms from the Robert Koch-Institute (RKI)](https://zenodo.org/records/14562231)
